# Effect of 2‐methoxyestradiol treatment on early‐ and late‐stage breast cancer progression in a mouse model

**DOI:** 10.1002/cbf.3842

**Published:** 2023-08-30

**Authors:** Kimberly T. Peta, Chrisna Durandt, Marlene B. van Heerden, Anna M. Joubert, Michael S. Pepper, Melvin A. Ambele

**Affiliations:** ^1^ Department of Immunology, Institute for Cellular and Molecular Medicine; South African Medical Research Council Extramural Unit for Stem Cell Research and Therapy; Faculty of Health Sciences University of Pretoria Arcadia South Africa; ^2^ Department of Oral and Maxillofacial Pathology, School of Dentistry, Faculty of Health Sciences University of Pretoria Pretoria South Africa; ^3^ Department of Physiology, School of Medicine, Faculty of Health Sciences University of Pretoria Pretoria South Africa

**Keywords:** 2‐methoxyestradiol, breast cancer, in vivo, metastasis, tumour growth

## Abstract

The prevalence of breast cancer (BC) continues to increase and is the leading cause of cancer deaths in many countries. Numerous in vitro and in vivo studies have demonstrated that 2‐methoxyestradiol (2‐ME) has antiproliferative and antiangiogenic effects in BC, thereby inhibiting tumour growth and metastasis. We compared the effect of 2‐ME in early‐ and late‐stage BC using a transgenic mouse model—FVB/N‐Tg(MMTV‐PyVT)—of spontaneously development of aggressive mammary carcinoma with lung metastasis. Mice received 100 mg/kg 2‐ME treatment immediately when palpable mammary tumours were identified (early‐stage BC; Experimental group 1) and 28 days after palpable mammary tumours were detected (late‐stage BC; Experimental group 2). 2‐ME was administered via oral gavage three times a week for 28 days after initiation of treatment, whereas control mice received the vehicle containing 10% dimethyl sulfoxide and 90% sunflower oil for the same duration as the treatment group. Mammary tumours were measured weekly over the 28 days and at termination, blood, mammary and lung tissue were collected for analysis. Mice with a tumour volume threshold of 4000 mm^3^ were killed before the treatment regime was completed. 2‐ME treatment of early‐stage BC led to lower levels of mammary tumour necrosis, whereas tumour mass and volume were increased. Additionally, necrotic lesions and anti‐inflammatory CD163‐expressing cells were more frequent in pulmonary metastatic tumours in this group. In contrast, 2‐ME treatment of late‐stage BC inhibited tumour growth over the 28‐day period and resulted in increased CD3+ cell number and tumour necrosis. Furthermore, 2‐ME treatment slowed down pulmonary metastasis but did not increase survival of late‐stage BC mice. Besides late‐stage tumour necrosis, none of the other results were statistically significant. This study demonstrates that 2‐ME treatment has an antitumour effect on late‐stage BC, however, with no increase in survival rate, whereas the treatment failed to demonstrate any benefit in early‐stage BC.

## Significance statement

- 2‐methoxyestradiol (2‐ME) is a synthetic oestrogen that has been shown to have antiproliferative and antiangiogenic effects in breast cancer (BC).
- In early‐stage BC, 2‐ME treatment did not have an antitumour effect.
- In late‐stage BC, 2‐ME treatment resulted in a decrease in tumour volume, mass and tumour progression.
- Tumour necrosis was significantly higher in the 2‐ME‐treated group in late‐stage BC. However, 2‐ME treatment did not increase overall survival.
- The antitumour effect of 2‐ME in late‐stage BC may be due to the drug's ability to induce tumour necrosis and increase the number of CD3+ T cells.
- 2‐ME may be a potential treatment option for late‐stage BC, but further studies are needed to confirm these findings and to optimize the dosing and delivery of the drug.

## 1 INTRODUCTION

Female breast cancer (BC) incidence has surpassed lung cancer with about 2.3 million new cases in 2020.^1^ BC incidence is based on new cases documented in 159 of 185 countries and is the leading cause of death in 110 countries.^1^ Although current treatments increase the survival rate, they target both cancer and healthy cells.^2^, ^3^ Moreover, some of these treatments may be ineffective for late‐stage metastatic BC.^3^ Given the limitations of current, other therapies have been investigated. One such therapy is a promising anticancer agent called 2‐methoxyestradiol (2‐ME), a natural endogenous steroid that is a metabolite of 17β‐estradiol (E2).^4^, ^5^, ^6^ E2 is generated by O‐methylation of estradiol at 2‐position and sequential hepatic hydroxylation.^6^, ^7^ 2‐ME is antiangiogenic and antiproliferative, and induces apoptosis of actively dividing cells in vitro and in vivo.^8^, ^9^ The apoptotic nature of 2‐ME effectivity extends to oestrogen‐independent and oestrogen‐dependent cell lines.^10^ 2‐ME targets dividing cells during the mitosis (G2/M) cell cycle phase and spares quiescent cells.^11^, ^12^ This drug binds to the colchicine‐binding site on tubulin, inducing microtubule depolymerization and inhibiting microtubule assembly,^13^, ^14^ consequently inhibiting proliferation and inducing apoptosis.^15^ However, 2‐ME does not impact the extent of tubulin assembly but impedes the rate.^16^

Due to the antiangiogenic and antiproliferative effects of 2‐ME, numerous studies have investigated its effect on BC. Many in vitro studies have demonstrated that 2‐ME inhibits tumour initiation, tumour growth and metastasis, and induces apoptosis in various BC cells in a dose‐dependent manner.^17^, ^18^, ^19^, ^20^ This is achieved by inhibiting microtubule turnover, which leads to cell cycle arrest and apoptosis.^7^, ^21^ Furthermore, 2‐ME decreases cell viability with increased 2‐ME concentrations and exposure time.^17^ LaVallee et al.^22^ exposed the MDA‐MB‐231 BC cell line to 2‐ME analogues and found that the analogues induced G2‐M cell cycle arrest and apoptosis after 4–16 h and 16–24 h respectively. Many in vivo studies have also demonstrated the antitumour effect of 2‐ME.^14^, ^18^, ^22^, ^23^, ^24^ However, some studies have suggested that the antiproliferative effect of 2‐ME is limited.^17^, ^18^ These studies suggested that 2‐ME may not inhibit but rather slow the rate of tumour growth, and that if 2‐ME is administered for a longer time before tumours appear, it may increase tumour growth.^17^, ^18^ Other studies suggested that the lack of antitumour activity may be due to suboptimal 2‐ME concentrations, which exhibit stimulatory effects, but not the inhibitory effect of 2‐ME, which is dose dependent.^17^, ^23^ The treatment dosages varied from 20 to 150 mg/kg given for varying numbers of days. All these studies have xenograft models, except for one allograft study where C3(1)/Tag transgenic mice developed spontaneous oestrogen receptor‐negative mammary carcinoma and were treated with 150 mg/kg/day with 2‐ME for 6 weeks.^18^ Treatment was given orally for two different periods before tumours formed at 12 weeks and after 18 weeks of age when palpable tumours were 0.5 cm in diameter.^18^ 2‐ME decreased tumour growth and burden in both treatment periods.^18^

A xenograft study with a similar treatment design, whereby 2‐ME treatment at a concentration of 150 mg/kg/day was given orally for 33 days when tumours reached 0.5 cm in diameter, revealed that 2‐ME inhibited angiogenesis and tumour growth from implanted MBA‐MB‐231 cell lines.^23^ This study showed that a higher concentration of 2‐ME given for a longer period can induce antitumour effects. However, a higher concentration (150 mg/kg/day) did not always result in antitumour activity. In another xenograft study, 2‐ME was given intraperitoneally (IP) and orally at 150 and 75 mg/kg/day, respectively, for 19 days after palpable tumours had developed. Treatment with 2‐ME showed no antitumour activity but, instead, increased tumour growth in mice inoculated with oestrogen receptor‐negative MBA‐MB‐435 cells and oestrogen‐dependent MCF‐7 cells.^25^ Klauber et al.^14^ suggested an optimal concentration of 75 mg/kg/day is needed to avoid toxic effects such as weight loss, diarrhoea, hair loss and lethargy. Despite the contradicting reports, most studies have clearly shown that prolonged administration of 2‐ME renders an antitumour activity after palpable tumours have developed.

Cytokines are involved in various stages of BC and play a crucial role in either inhibiting or stimulating BC invasion and proliferation.^26^, ^27^ Interferons, interleukins (IL) such as IL‐12 and IL‐18 inhibit BC, whereas IL‐6, IL‐1, transforming growth factor β and IL‐11 stimulate BC.^26^ These cytokines are secreted by immune cells such as macrophages and T cells contributing to the inflammatory tumour microenvironment (TME).^27^, ^28^ BC cells secrete factors that differentiate macrophages toward the M2 phenotype.^29^ M2‐associated CD163+ macrophages are a prognostic marker for BC and metastasis and an increased number of CD163+ macrophages are associated with decreased patient survival.^30^, ^31^, ^32^ In contrast, higher levels of CD3+ cells are associated with good prognosis biomarkers such as CD8 and CD20, and are associated with increased survival.^33^, ^34^, ^35^

Many studies have demonstrated an antitumour effect of 2‐ME on BC progression, most of which involve xenograft models. However, no study has investigated the distinct effect of 2‐ME treatment on early‐ and late‐stage BC progression. In this study, a transgenic mouse model (FVB/N‐Tg(MMTV‐PyVT)634Mul/J) that spontaneously develops mammary tumours with lung metastasis (exhibiting an aggressive phenotype of BC)^36^ was used to investigate and compare the effect of 2‐ME treatment on early‐ and late‐stage BC progression. This mouse model can effectively replicate the progression of mammary gland tumours, closely resembling the stages observed in human ductal BC. The tumours displayed gene expression patterns consistent with luminal B subtype human BC and they also exhibited shared histopathological features and the expression of basal‐like markers, mirroring the characteristics of aggressive basal‐like BC in humans. Furthermore, we delved into the cytokine profile and explored immunohistochemistry (IHC) of key prognostic biomarkers.

## 2 MATERIALS AND METHODS

### 2.1 Animal studies

This study was approved by the Faculty of Health Sciences research ethics committee (ethics reference no.: REC166‐19) and the animal ethics committee (ethics reference no.: 534/2019) of the University of Pretoria. The FVB‐TgN(MMTV‐PyVT) mouse model was obtained from the Jackson Laboratory and mice were bred to obtain heterozygous offspring by crossing hemizygous males with wild‐type females. All offspring were genotyped and only heterozygous females were included in the study.

### 2.2 Animal genotyping

Mouse genotyping was performed using the KAPA Mouse Genotyping Kit (KAPABIOSYSTEM) according to the manufacturer's instructions. Briefly, a 2 mm mouse tail biopsy was placed in 0.2 mL microcentrifuge tubes and DNA was extracted. For polymerase chain reaction (PCR) genotyping experiments, two pairs of primer sequences obtained from the Jackson Laboratory website (The Jackson Laboratory) were used. The forward primer 5′‐GGAAGCAAGTACTTCACAAGGG‐3′ and reverse primer 5′‐GGAAAGTCACTAGGAGCGGG‐3′ were specific for the transgene and the forward primer 5‐′CAAATGTTGCTTGTCTGGTG‐3′ and reverse primer 5′‐GTCAGTCGAGTGCACAGTTT‐3′ were specific for internal positive control. A thermocycler (GeneAmp® PCR System 9700) was used to amplify DNA under the following conditions: initial denaturation at 95°C for 3 min, denaturation at 95°C for 15 s, annealing at 60°C for 15 s and extension for 15 s for 2 min for 35 cycles. The sizes of the amplicons were determined using ethidium bromide‐stained 2% agarose gel electrophoresis.

### 2.3 2‐ME treatment and tumour measurements

Treatment was divided into two experimental groups: one for early‐stage BC and the other for late‐stage BC. The early‐stage BC (Ex. 1) treatment commenced immediately when palpable mammary tumours were felt. The late‐stage BC (Ex. 2) treatment began 28 days after palpable tumours were felt. In both experiments, mice received 100 mg/kg of 2‐ME in a vehicle made up of 90% sunflower oil (Sunfoil) and 10% dimethyl sulfoxide given three times per week via oral gavage for 4 weeks followed by killing of animals. The control mice received the vehicle. Late‐stage BC mice on average received treatment eight times in both (control and treatment) groups. Mice that reached the mammary tumour volume threshold of ~4000 mm^3^ were terminated to avoid suffering as a result of tumour burden. Mice in the early‐stage BC group received 2‐ME treatment a total of 12 times.

A 2‐ME concentration of 100 mg/kg administered three times a week was chosen based on literature, to avoid adverse effects in mice. During the duration of the treatment, palpable mammary tumours were measured once a week and at termination using a calliper. Tumour volume was calculated using the formula *L* × *W* ^2^/2, where *L* is the length and *W* is the width.^37^ At termination, mammary tumours were excised and the mass was measured in grams on a scale (Sartorius). A light microscope (OLYMPUS, SC 100) was used to identify and physically count the number of metastatic lesions on the surface of the lungs. Images were also captured using the CellSens dimension imaging software (XV Imaging, product version 3.9). A total of 18 heterozygous female mice (nine for 2‐ME and nine for control) were used for each of the experimental procedures.

### 2.4 Histology and IHC analysis

Lung and mammary tissues were collected from killed 2‐ME‐treated and control group mice, and were fixed in 10% neutral buffered formalin. Haematoxylin and eosin (H&E) staining was performed as previously described.^38^, ^39^ IHC analysis for CD163 and CD3 was also performed according to previously described protocols^38^, ^39^ with slight modifications. Briefly, formalin‐fixed paraffin‐embedded tissue blocks were cut into 3 μm sections and baked in a 58°C oven overnight. Xylene was used to deparaffinize slides, whereafter they were hydrated with decreasing concentrations of alcohol to distilled water. A 3% hydrogen peroxide solution was used to quench endogenous peroxidase for 5 min at 37°C. Antigen retrieval was performed using a high pH buffer retrieval solution (Dako Envision FLEX Retrieval solution high pH, Agilent Technologies), washed in phosphate‐buffered saline (PBS) and background staining was subsequently blocked with a protein block (Novolink Leica Biosystems) for 30 min at room temperature (RT). The sample sections were incubated overnight at 4°C in a 1:300 anti‐CD163 antibody (EPR19518) (ab182422) (Abcam) and washed in PBS. Detection of the antigen–antibody binding site was performed with Novolink^TM^ Polymer Detection Kit (Leica Biosystems) as recommended by the manufacturer. Slides were rinsed in PBS and incubated with 3,3′‐Diaminobenzidine (DAB) (Novolink^TM^ Polymer Kit) for chromogen detection. Sections were washed and counterstained in haematoxylin for 1 min, dehydrated with increasing concentration alcohol solutions, cleared in xylene and mounted with dibutylphthalate polystyrene xylene. CD3 IHC was performed on 3 μm sections in a manner similar to CD163 but with a few differences. The antigen was retrieved using a low pH buffer (Cell Conditioning Solution CC2, Ventana Medical Systems, Inc.). Sections were incubated in a 1:100 rabbit monoclonal anti‐CD3 (Abcam ab16669 clone SP7) antibody for 120 min. Slides were rinsed in PBS and detected for 30 min at RT with antirabbit Polymer HRP IgG (Novolink^TM^ Polymer Detection Kit, Leica Biosystems). The negative controls were prepared by staining with PBS instead of CD163 or CD3 antibody. The Leica AT 2 Aperio scanner (Leica Biosystems) was used to capture images at ×40 magnification and Qupath software (https://github.com/qupath/qupath/releases/tag/v0.3.2), version 0.3.2 (The Queens University of Belfast) was used for analyses. Each scanned tissue section was imported into the software and the image type was set to brightfield (H‐DAB). A boundary was drawn around tissue section limits to focus only on the cells within the section. To distinguish between positive and negative cells, the estimation stain vector was set to automatic. The software was trained by identifying positively stained cells and indicating to the software what was viewed as positive cell detection. The detection image parameter was set to ‘optical density sum’ and the scan was initiated. After completion, the result showed the number of positive, negative and total cell counts. The number of positive cells was divided by the total number of cells to obtain the percentage of CD163‐ and CD3‐positive cells.

### 2.5 Measurement of plasma cytokines

Mice were killed with Isoflurane (Isofor; Piramal I Healthcare) and blood (800 µL) was collected via cardiac puncture into EDTA tubes and centrifuged at 28,487*g* for 15 min. Plasma (250–300 µL) was aliquoted in 2 mL microcentrifuge tubes. For cytokine profiling, the Legendplex mouse inflammation panel (13‐plex) kit (Biolegend®) was used. The kit tested for 13 mouse cytokines, which are monocyte chemoattractant protein*‐*1 (MCP‐1), granulocyte‐macrophage colony*‐*stimulating factor (GM‐CSF), tumour necrosis factor‐α (TNF‐α), interferon‐γ (IFN‐γ), IFN‐β, IL‐1α, IL‐1β, IL‐6, IL‐10, IL‐12p70, IL‐17A, IL‐23 and IL‐27. The assay was performed according to the manufacturer's instructions. Briefly, increasing standard concentrations (standard supplied with kit) were prepared (in duplicate) in a 96‐well plate and data acquired on a Cytoflex flow cytometer (Beckman Coulter). A standard curve was generated. The plasma samples were diluted twofold and staining was performed according to the manufacturer's instructions. The samples were analysed using a Cytoflex flow cytometer and the respective cytokine concentrations were calculated using Biolegend's data analysis software https://legendplex.qognit.com/.

### 2.6 Statistical analysis

GraphPad Prism version 5 (GraphPad Software Inc.) was used for statistical analysis. To compare the means between two groups, a one‐tailed unpaired *t* test was used. Data were presented as mean ± SEM. To compare the means of more than two categories, a multiple comparison test and a two‐way analysis of variance was used.

## 3 RESULTS

### 3.1 Distinct effects of 2‐ME treatment on tumour volume and mass in early‐ and late‐stage BC

**Figure 1:**
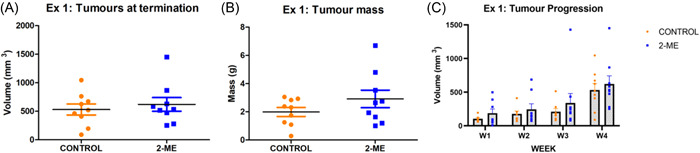
2‐Methoxyestradiol (2‐ME) treatment of early‐stage breast cancer. (A) Average tumour volumes were the same in both groups at termination (*p* = .2847). (B) The average tumour mass was higher in the 2‐ME‐treated group (*p* = .1004). (C) Throughout the 4 weeks, tumour volumes of 2‐ME‐treated mice were higher (*N* = 9 in each group).

**Figure 2:**
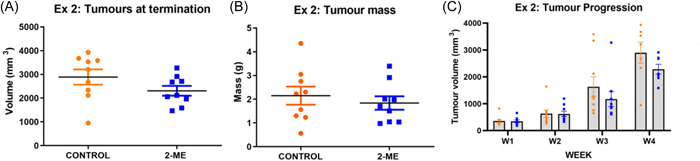
2‐Methoxyestradiol (2‐ME) treatment of late‐stage breast cancer. (A) Tumour volume (*p* = .0729) and (B) tumour mass (*p* = .2624) were higher in the control group. (C) Equivalent tumour volumes were observed in weeks 1 and 2 but higher tumour volumes were observed in the control group in weeks 3 and 4 (*N* = 9 in each group).

Heterozygous female mice were genotyped and those that had the MMTV‐PyVT transgene were included in the study (Supporting Information: Figure 1). Tumour mass is the measure of tumour weight and tumour volume is the calculated volume based on tumour diameter.^40^ In early‐stage BC (Ex. 1), there was an increase in tumour mass in the 2‐ME‐treated mice, whereas there was no statistically significant difference in tumour volumes between both groups at termination (Figure 1A,B). The mice in the 2‐ME‐treated mice group had continuously higher tumour volumes throughout the 4‐week period, although this was not statistically significant (Figure 1C). In late‐stage BC (Ex. 2), tumour volume and mass were lower in the 2‐ME group at termination (Figure 2A,B). Tumour volumes of both groups were similar at weeks 1 and 2; however, in weeks 3 and 4, lower tumour volumes were observed in the 2‐ME group (Figure 2C), although this was not statistically significant.

### 3.2 2‐ME induces higher pulmonary lesion count in early‐stage BC and shows no statistical difference in late‐stage BC

**Figure 3:**
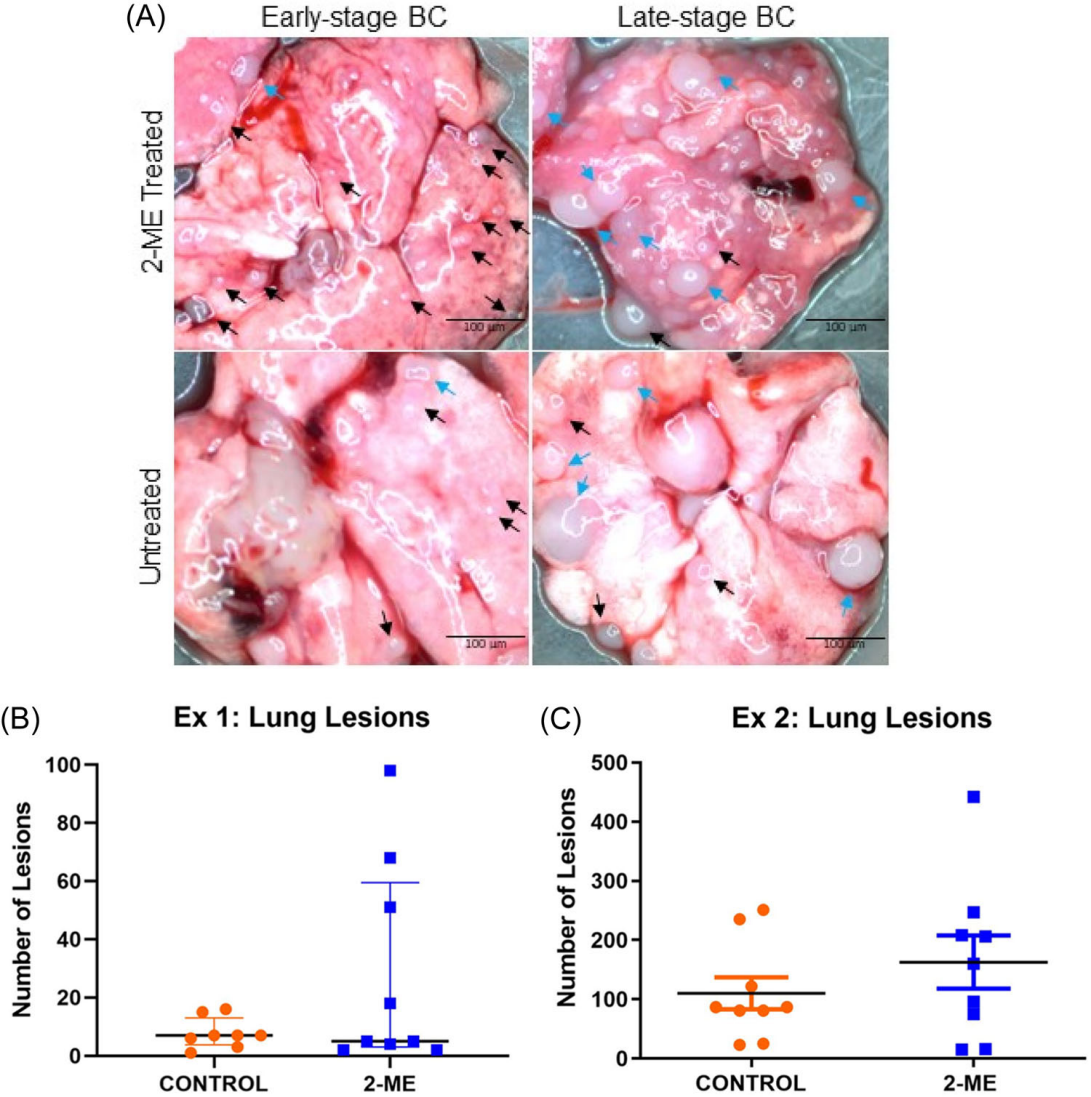
(A) Pulmonary lesions of early‐stage breast cancer (BC) were smaller (arrows) and a combination of small (black) and large (blue) lesions were observed in late‐stage BC. (B) A greater number of pulmonary lesions were observed in both the early‐stage (*p* = .1169) and (C) late‐stage (*p* = .1654) 2‐methoxyestradiol (2‐ME)‐treated mice (*N* = 9 in each group).

Mouse lungs were examined for pulmonary lesions at termination. Pulmonary lesions, which appeared as nodules, were identified and counted. The lesions in early‐stage BC were smaller compared with late‐stage BC lesions, which were larger with varying sizes (Figure 3A). In early‐stage BC‐treated mice, the number of pulmonary lesions was higher in the 2‐ME group compared with the control group with a *p*‐value of 0.0654 (Figure 3B). Likewise, in late‐stage BC‐treated mice, a greater number of pulmonary lesions were observed in the 2‐ME‐treated mice (Figure 3C), although this was not statistically significant.

### 3.3 Differential impact of 2‐ME on tumour necrosis: Reduced mammary tumour necrosis in early‐stage BC and statistically higher necrosis in late‐stage BC

**Figure 4:**
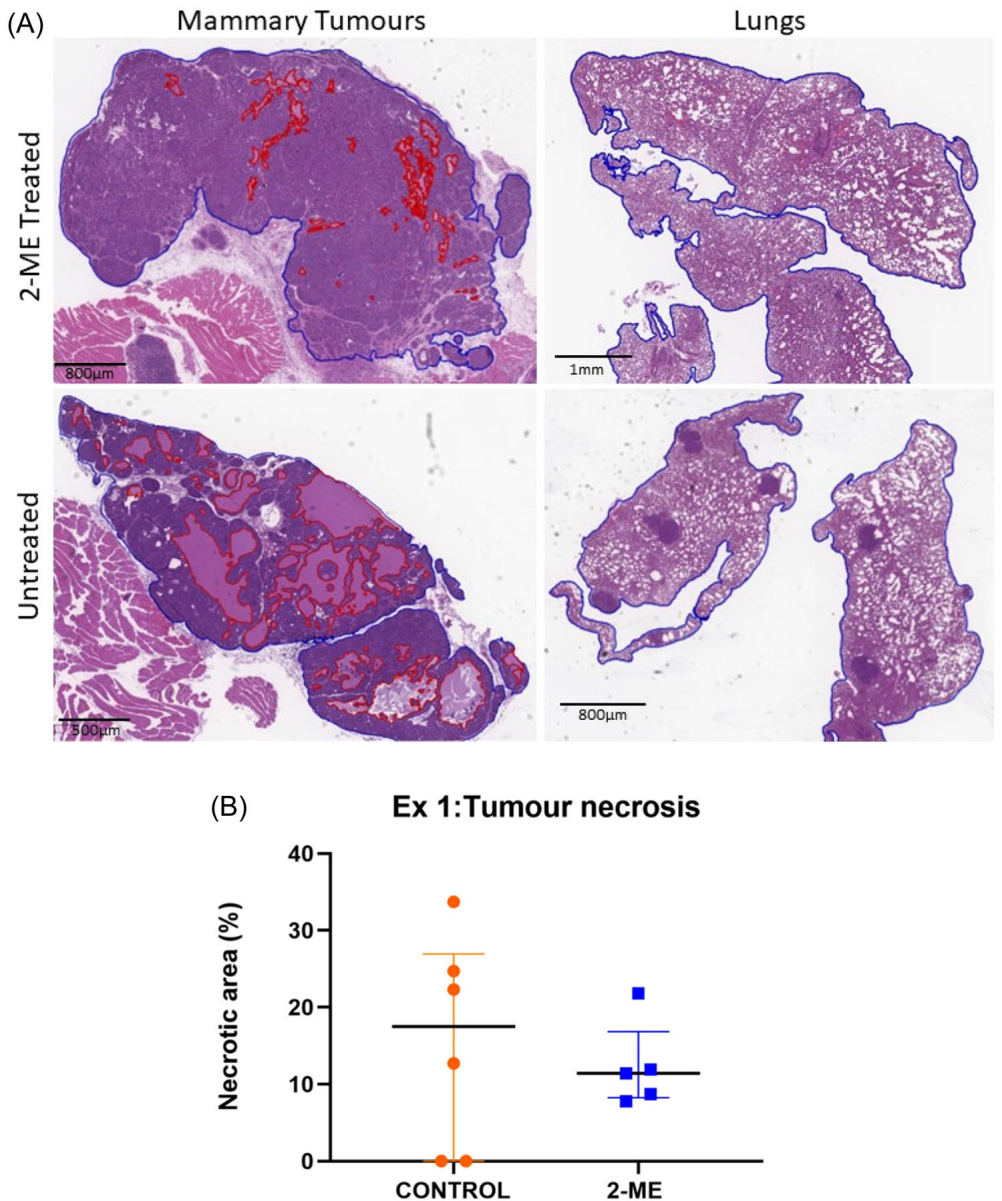
Histopathological analysis of mammary tumour and lungs in early‐stage breast cancer mice treated with 2‐methoxyestradiol (2‐ME). (a) Early‐stage tumour and pulmonary tissues labelled with red boundaries represent necrotic regions. (B) Greater tumour necrosis (*p* = .3176) was observed in the control group compared with the 2‐ME group (Control *N* = 6, 2‐ME *N* = 5).

**Figure 5:**
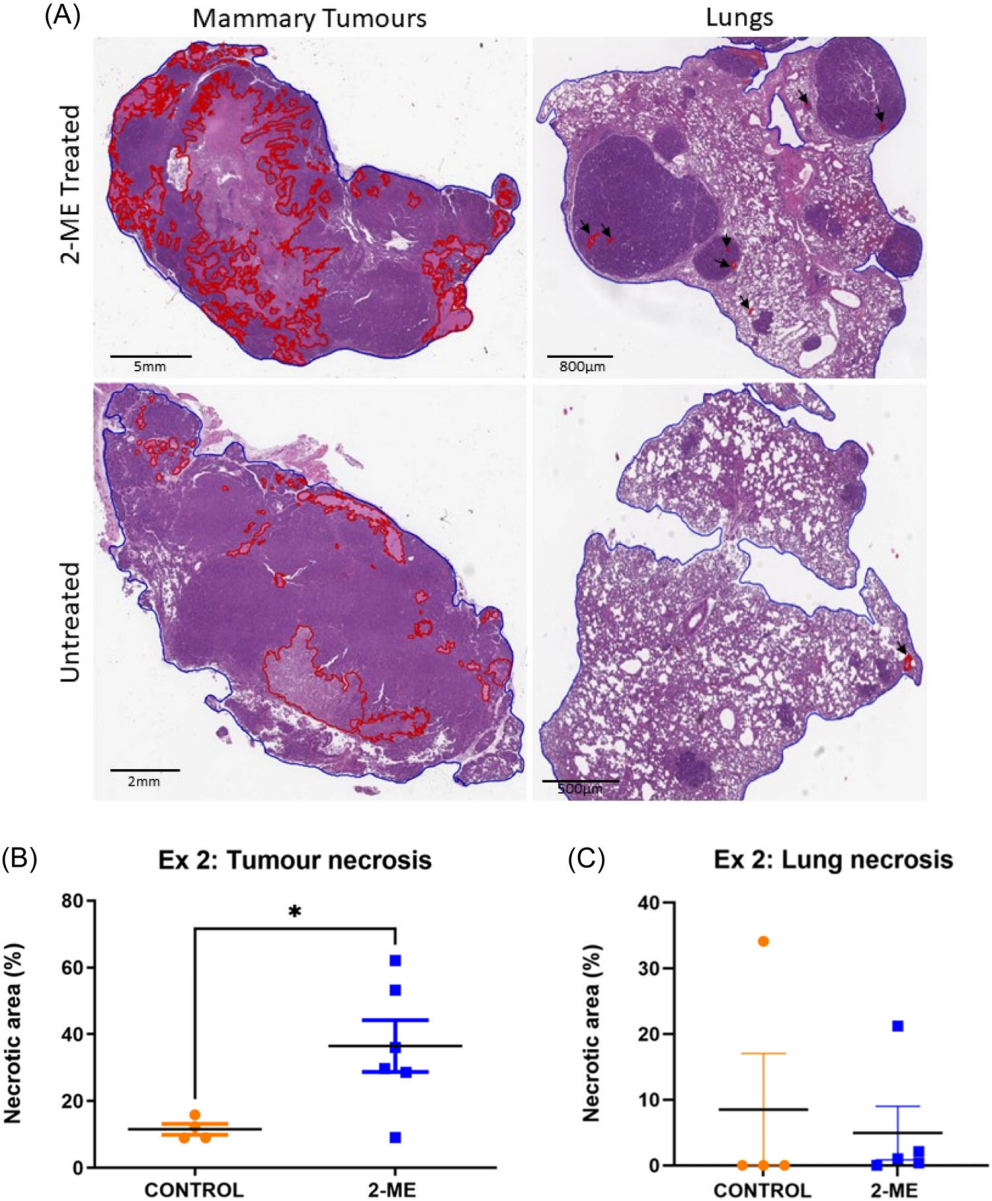
Histopathological analysis of mammary tumour and lung tissues from late‐stage breast cancer mice treated with 2‐methoxyestradiol (2‐ME). (A) Red boundaries surrounding necrotic areas of late‐stage mammary tumours and pulmonary tissues are shown with arrows. (B) Tumour necrosis was significantly higher in the 2‐ME‐treated mice, *p* = .0169 (Control *N* = 4, 2‐ME *N* = 6). (C) Control group pulmonary necrosis (*p* = .3480) was higher than in the 2‐ME group (Control *N* = 4, 2‐ME *N* = 5).

The H&E images taken were analysed by drawing red lines around the necrotic regions and a blue line around the entire tissue (Figure 4A). No necrotic pulmonary regions were observed for early‐stage BC. To calculate the proportion of necrotic tissue, the total sum of the necrotic regions was divided by the area of the entire tissue. Mammary tumour necrosis was lower in the 2‐ME group in early‐stage BC (Figure 4B). Only one mouse in the 2‐ME group had pulmonary necrosis. In late‐stage BC, tumour necrosis was significantly (*p* = .0169) higher in the 2‐ME group than in the control group (Figure 5A,B). Pulmonary necrosis was lower in the 2‐ME group, albeit not significant (Figure 5C).

### 3.4 Distinct modulation of CD163 + M2 macrophages by 2‐ME: Fewer numbers in early‐stage BC mammary tumours but increased counts in lung tissue and no significant differences in late‐stage BC

**Figure 6:**
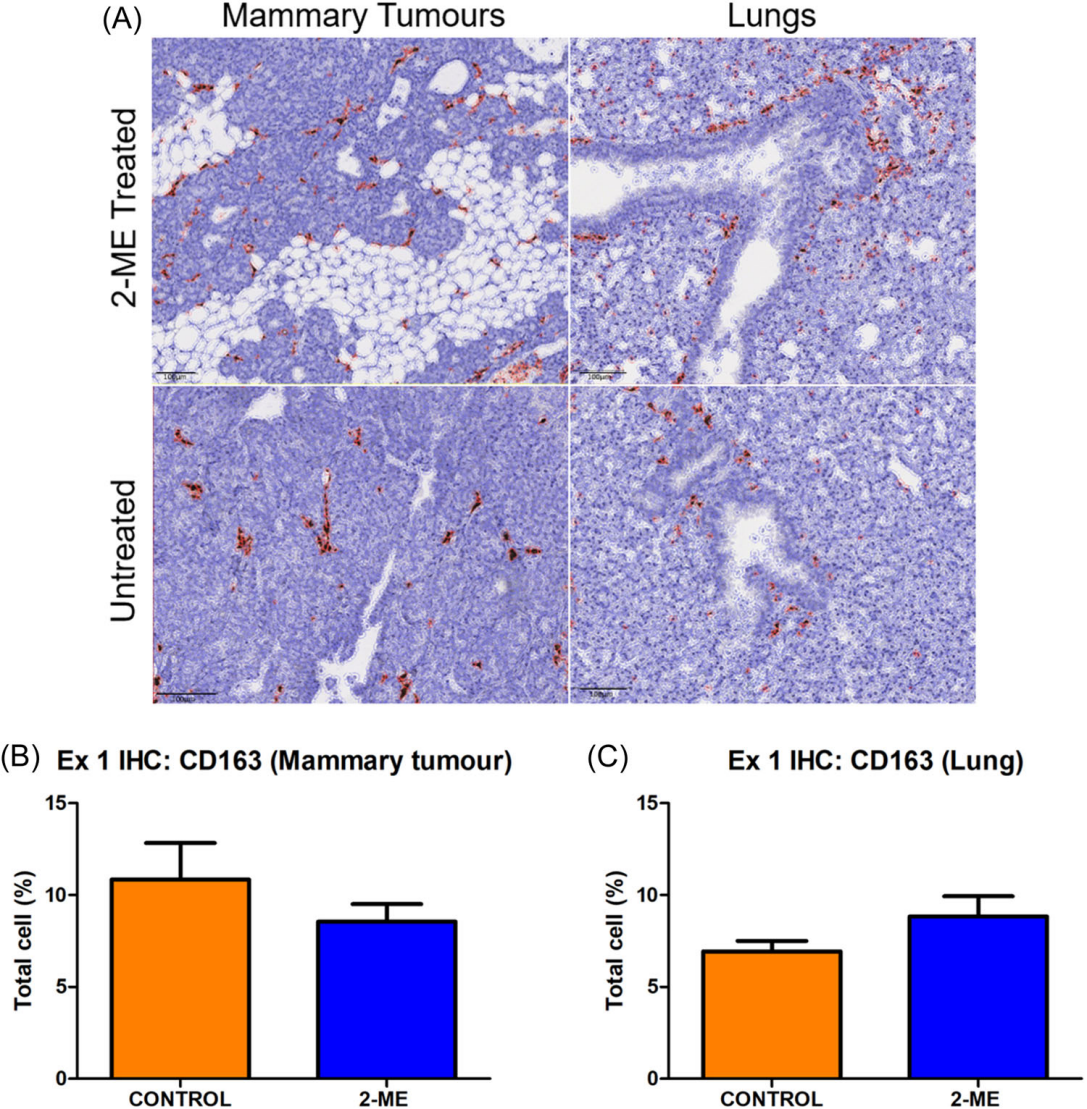
Immunohistochemistry (IHC) for CD163 staining in mammary tumour tissue and lung of early‐stage breast cancer treated with 2‐methoxyestradiol (2‐ME). (A) In early‐stage mammary and lung tissue, brown staining represents CD163+ macrophages. (B) Early‐stage 2‐ME‐treated mice had a lower number (*p* = .1617) of CD163+ macrophages in mammary tissue and (C) a higher number (*p* = .0811) of CD163+ macrophages in lung tissue (*N* = 5 in each group).

**Figure 7:**
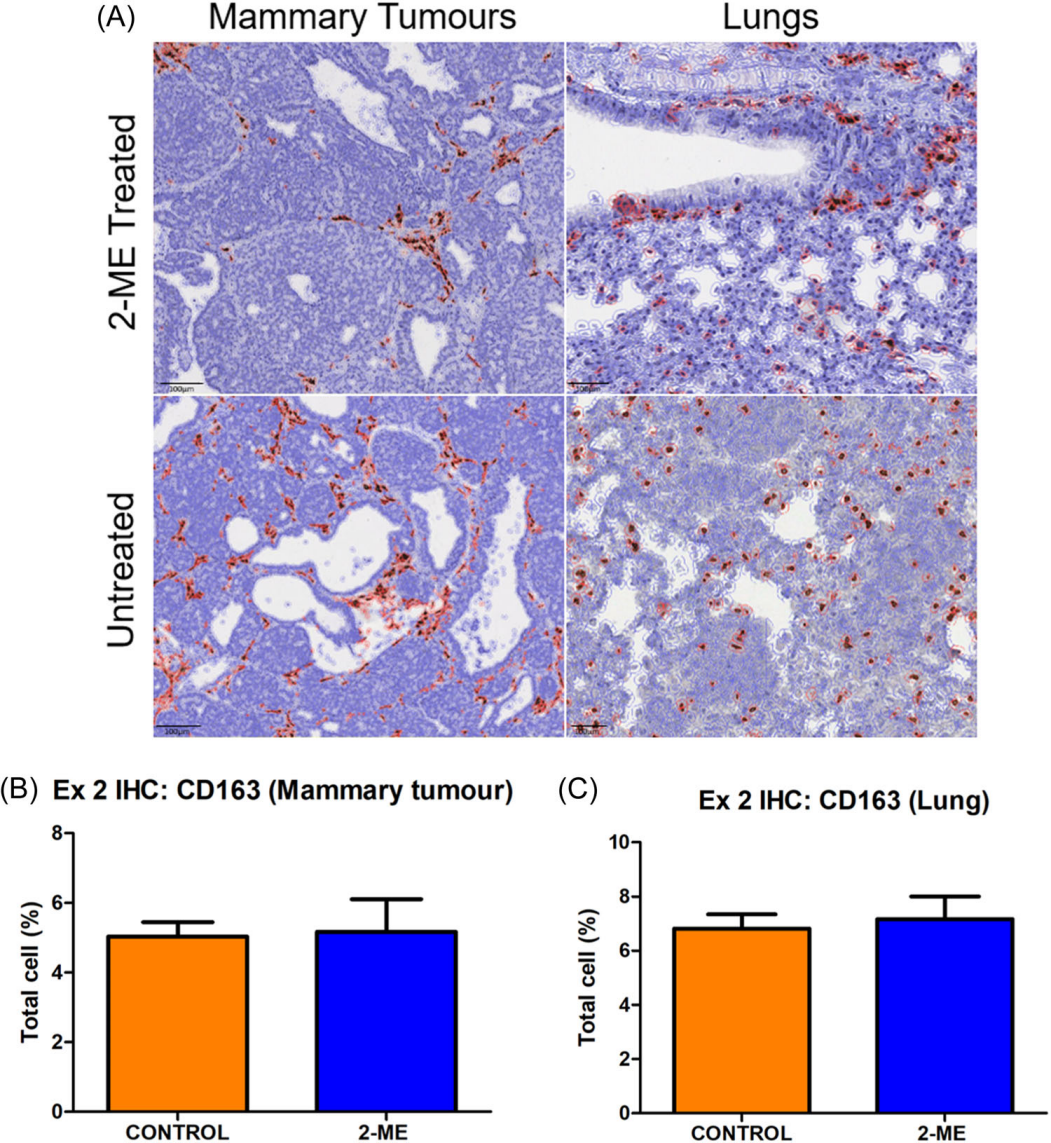
Immunohistochemistry (IHC) for CD163 staining (brown‐stained cells) in mammary tumour tissue and lung of late‐stage breast cancer treated with 2‐methoxyestradiol (2‐ME). (A) Late‐stage mammary and lung tissue IHC. (B) A similar number of CD163+ macrophages was noted in the mammary (*p* = .4546) and (C) lung tissues in both groups (*p* = .3729) (Control *N* = 3 and 2‐ME *N* = 5).

Dark brown stains represent CD163+ macrophages (Figure 6A). In early‐stage BC, the number of CD163+ macrophages in 2‐ME‐treated mice was lower in mammary tumour tissues but higher in lung tissue (Figure 6B,C). In late‐stage BC, there was no difference in the number of CD163+ macrophages between the 2‐ME‐treated and the control group both in mammary and lung tissues (Figure 7A–C).

### 3.5 CD3+ T cell distribution in response to 2‐ME revealed no significant differences in early‐stage BC, but increased counts in late‐stage BC mammary tumours and decreased counts in lung tissue

**Figure 8:**
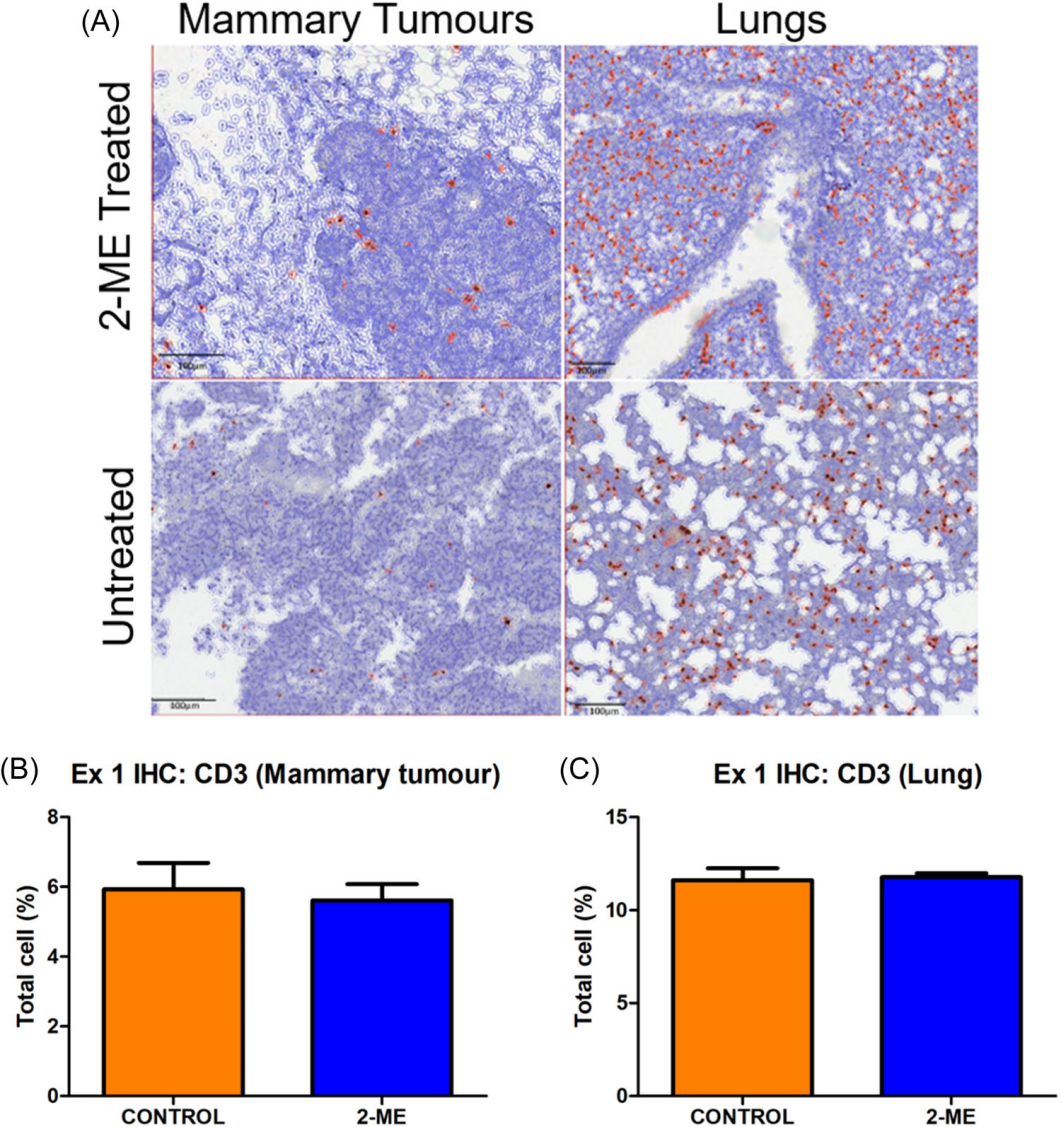
(A) CD3+ T cells are stained brown and encircled in red. (B, C) No difference was observed in early stage (*p* = .3665) for breast cancer for both mammary tumours and pulmonary tissue (*p* = .4040). (Control *N* = 3 and 2‐methoxyestradiol [2‐ME] *N* = 5).

**Figure 9:**
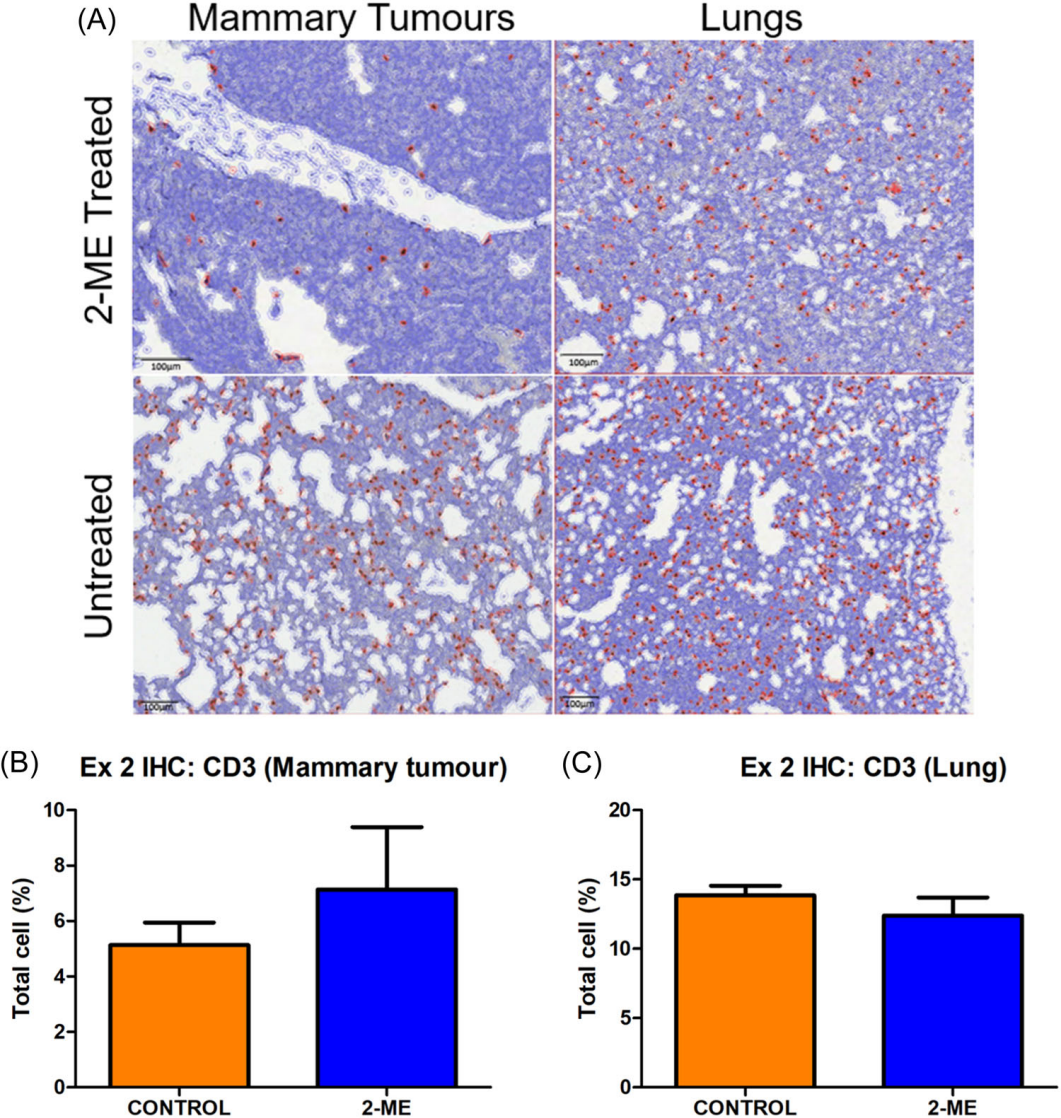
(A) CD3+ T cells are encircled in red. (B) In early‐stage breast cancer, a higher number of CD3+ cells were observed in mammary tumours (*p* = .3018) and (C) fewer CD3+ cells were detected in the pulmonary tissue (*p* = .2243) of 2‐methoxyestradiol (2‐ME)‐treated mice (Control *N* = 3 and 2‐ME *N* = 5).

Stained (brown) cells represent CD3+ T cells (Figures 8A and 9A). No difference was observed between groups for mammary tumours and pulmonary metastasis in early‐stage BC (Figure 8B,C). However, in late‐stage BC, the number of CD3+ T‐cell was higher in the mammary tumours, but lower in the lung tissue of 2‐ME‐treated mice (Figure 9B,C). None of the results were significant.

### 3.6 Late‐stage BC survival analysis revealed the 2‐ME group showed lower survival and reduced tumour volumes at termination

**Figure 10:**
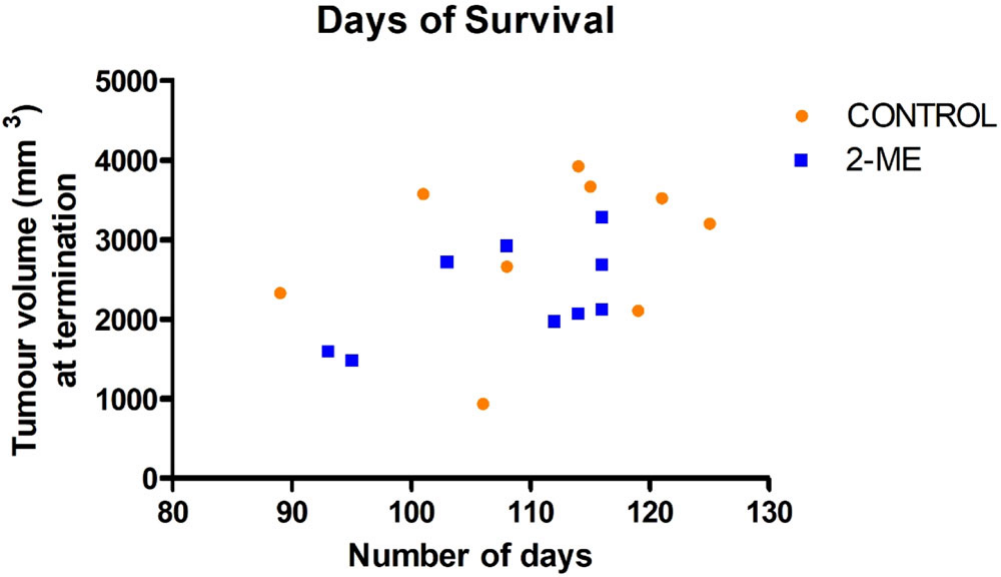
Days of survival of late‐stage breast cancer mice. At termination, the number of days of survival of mice in the control group exceeded that of the 2‐methoxyestradiol (2‐ME) treatment group (*N* = 9).

The number of days mice in the late‐stage BC experimental group lived before termination was evaluated to assess the effect of 2‐ME on survival. Importantly, mice with mammary tumours that reached a volume of ~4000 mm^3^ were terminated due to heavy tumour burden that impaired quality of life. The decision to terminate was determined by a qualified veterinarian. On average, mice in the 2‐ME group lived for fewer days compared to mice in the control group (Figure 10). However, as previously stated, the tumour volumes of the 2‐ME group were lower at the point of termination.

### 3.7 Cytokine concentrations associated with early‐stage BC

**Figure 11:**
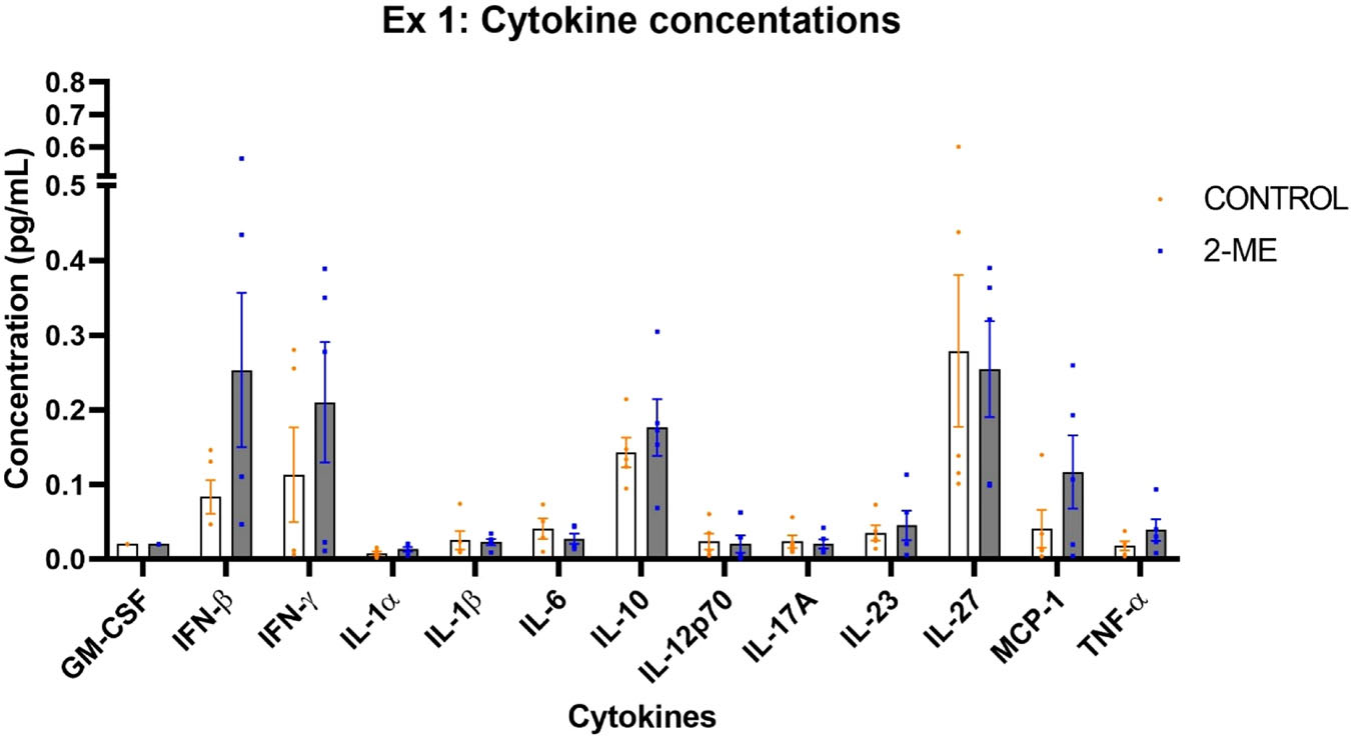
Plasma cytokine concentrations of the control and 2‐methoxyestradiol (2‐ME) (Ex. 1) groups: Cytokine concentrations that were notably higher in the 2‐ME group were interferon (IFN)‐β, IFN‐γ, interleukin‐10 (IL‐10) and monocyte chemoattractant protein*‐*1 (MCP‐1), whereas IL‐6 and IL‐27 were lower. The other cytokines were present at equivalent levels in both groups (*N* = 5 in each group).

Cytokines associated with inflammation were measured in the plasma 2‐ME‐treated and untreated mice in early‐stage BC. The following cytokine concentrations were similar in both groups: IL‐1α, IL‐1β, IL‐12p70, IL‐17A and GM‐CSF. Cytokines that were higher in the 2‐ME‐treated group were IFN‐β, IFN‐γ, IL‐10, IL‐23, MCP‐1 and TNF‐α, with IFN‐β, IFN‐γ, IL‐10 and MCP‐1 notably high, but not statistically significant. IL‐6 and IL‐27 levels were lower in the 2‐ME group (Figure 11).

## 4 DISCUSSION

The effect of 2‐ME on early‐ and late‐stage BC was investigated using a transgenic mouse model that represents an aggressive form of spontaneous mammary carcinoma. The study aimed to simulate a clinical scenario in which 2‐ME treatment is given early (when a palpable tumour first appears) or late (28 days after a palpable mammary tumour first appears), depending on the stage at diagnosis. The mice were given 100 mg/kg 2‐ME treatment orally three times a week for 4 weeks. Thus, the total number of doses administered in the early‐stage BC group was 12. Due to the decision to terminate earlier due to excessive tumour burden in the late‐stage BC group, the total number of doses administered in both the 2‐ME‐treated and control groups was 8.

In early‐stage BC, mammary tumour volumes in the 2‐ME‐treated and control groups were the same, but tumour progression was more rapid and tumour mass increased. The latter observation is supported by the lower degree of mammary tumour necrosis seen in the 2‐ME‐treated group, as increased cancer cell necrosis indicates an antitumour effect.^41^ However, the number of CD163+ macrophages was lower in mammary tumours, which does not support the findings considering the phenotypic characteristics of higher tumour progression and increased tumour mass. The number of CD3+ T cells in the mammary TME was similar in both groups. These phenotypic results may account for similar tumour volumes at the point of termination, as large tumours are associated with fewer CD3+ T cells^42^ and CD163+ macrophages are associated with median‐sized tumours.^43^ Although mammary tumour volumes in the 2‐ME‐treated group were higher in the week preceding termination, this was not the case at termination. Due to the drug's prolonged exposure, it appears that 2‐ME inhibits tumour growth. Longer 2‐ME exposure has previously been shown to cause BC cell apoptosis,^17^ suggesting that if 2‐ME is given for a longer period it may have an antitumour effect. Furthermore, pulmonary lesions were more common in the 2‐ME‐treated group, as evidenced by a higher number of CD163+ macrophages detected, but there was no discernible difference in the number of CD3+ T cells. An in vivo study demonstrated that increased CD163+ macrophages led to enhanced metastatic ability and tumorigenicity,^32^ and we observed similar trends. Taken together, our findings suggest that 2‐ME did not cause an antitumour effect. A study by Huh et al.^18^ on the late intervention of 2‐ME, which correlates with the early‐stage BC group in this study, found that there was a 60% decrease in tumour volume in 2‐ME‐treated C3(1)/Tag transgenic mice compared with controls, and suggested that a high dose of 150 mg/kg can decrease tumour volume and inhibit angiogenesis. This result is contrary to what we found in this study and could be because of the increased dosage of 150 mg/kg/daily for 6 weeks. Similarly, another study reported that 2‐ME is antitumorigenic, but with a high dose of 150 mg/kg/day for 33 days.^21^ There are several other studies with a similar experimental design to the early‐stage group, that is, treatment was initiated after detecting the presence of palpable tumours. However, the dose and duration of treatment varied with the dose ranging from 50 mg/kg/day for 16 days to 75 mg/kg/day for 30 days.^14^, ^24^, ^44^ Despite the concentration variations, all studies reported an antitumour effect for 2‐ME. Oral administration of a concentration of 25 mg/kg/day has proven effective against metastasis.^45^ What stands out between previous studies and this study is that 2‐ME was administered daily in most studies as opposed to the approach used in this study, which used a spaced‐out treatment (thrice per week for 4 weeks). In hindsight, the dosage schedule should be reconsidered in future studies as it seems that antitumour effect of 2‐ME is based on its consistent bioavailability to the TME. This hypothesis is supported by a study on the pharmacokinetics of 2‐ME, which reported that the bioavailability of 2‐ME at 10 mg/kg was low after 24 h in the plasma of mice.^46^ Furthermore, the authors reported that oral administration of 20 mg/kg/day for 28 days showed no statistically significant effect on tumour growth.^46^ Taken together, the results suggest that 2‐ME should be given daily at a dose higher than 20 mg/kg/day to cause an antitumour effect.

Notably, 2‐ME‐treated mice had higher levels of the inflammatory cytokines, IFN‐β, IFN‐γ, IL‐10 and MCP‐1, whereas IL‐6 and IL‐27 were lower. Studies have shown that MCP‐1 is elevated in BC and has been implicated in BC progression.^27^, ^47^ Moreover, MCP‐1 is involved in cancer initiation and activates monocytes that promote pulmonary metastasis in BC.^48^, ^49^ Interferons are antitumourigenic and inhibit BC cells' capacity to form mammospheres.^50^, ^51^ IL‐6 and IL‐10 are anti‐inflammatory cytokines that are also protumorigenic.^27^, ^52^ Significantly elevated IL‐27 levels have been observed in BC patients and are associated with tumour growth.^53^ Even though elevated levels of interferons appear to indicate an antitumour effect, most of these other notable cytokines indicate that 2‐ME may have a pro‐tumour effect in early‐stage BC.

Contrary to early‐stage BC, the 2‐ME effect in late‐stage BC suggests antitumour activity. Tumour volume, mass and tumour progression were lower in the 2‐ME group. This observation was supported by tumour necrosis which was significantly higher in 2‐ME‐treated mice, as was the number of CD3+ T cells. CD3+ T‐cell number is associated with increased survival and patients with low levels of CD3+ T cells had an elevated risk of relapse in BC.^54^, ^55^ However, there was a similar number of CD163+ macrophages in mammary tumours of the 2‐ME‐treated and control groups. Furthermore, pulmonary lesions were higher and pulmonary necrosis was lower in the 2‐ME‐treated group. This phenotypic finding is further supported by the presence of fewer CD3+ T cells in the pulmonary tissue. In addition, despite having less pulmonary necrosis on average in the 2‐ME group, more mice in this group had pulmonary necrosis. This suggests that 2‐ME slowed down pulmonary metastasis which is supported by the lack of difference in the number of CD163+ macrophages observed between the 2‐ME and the control groups. Taken together, our data suggest that 2‐ME rendered an antitumour effect on mammary tumours, but not on pulmonary metastases. These results demonstrate that 2‐ME treatment is not effective in inhibiting metastasis in mice that receive treatment late. Generally, treating advanced BC with current therapies is challenging^56^, ^57^ and advanced BC treatments are aimed at prolonging and improving the quality of life.^58^, ^59^ Studies have shown that 2‐ME treatment inhibits tumorigenesis in advanced BC and increases the overall survival rate.^18^ We also observed an antitumour effect on late‐stage BC, but 2‐ME failed to increase overall survival. In previous studies, 2‐ME treatment was given to mice either on the day of inoculation,^24^ or after the appearance of palpable tumours.^14^, ^18^, ^21^

The use of 2‐ME as a treatment option comes with certain limitations. One significant drawback is its poor oral bioavailability, as it is not efficiently absorbed into the bloodstream after oral intake. This is due to extensive metabolism in the liver before reaching systemic circulation.^60^ Additionally, 2‐ME has a short half‐life, necessitating frequent administration to maintain therapeutic levels in the blood,^13^ and 2‐ME can induce side effects such as weight loss, lethargy, hair loss and diarrhoea.^14^ To mitigate some of the limitations as well as potential toxic effects of 2‐ME treatment, our study administered a dose of 100 mg/kg, three times a week to the mice, well below the 75 mg/kg threshold reported by Klauber et al.^14^ However, despite the higher dose, none of the mice experienced any toxicity from the 2‐ME treatment.^14^ Future study should consider IP administration of 2‐ME, which will allows for faster drug absorption into the bloodstream, thereby bypassing liver metabolism. However, we were faced with a great challenge regarding the solubility of 2‐ME in solvents suitable for IP administration in our study as the 2‐ME used could only be dissolved in hydrophobic substances, thereby making IP delivery unfeasible. To bypass this, researchers are investigating novel 2‐ME analogues.^13^

To our knowledge, this is the first in vivo study that used this late‐stage BC treatment strategy and at the same with the early‐stage BC for accurate comparison. Further studies are needed to understand the effect of 2‐ME on advanced mammary carcinoma from a mechanistic perspective. Furthermore, the role of 2‐ME in the TME, the pharmacokinetic profile of the drug and its effect on leucocytes should be investigated. This could lead to the development of optimal 2‐ME treatment strategies capable of eliminating BC cells at every stage.

## 5 CONCLUSION

Our data suggest that 2‐ME has the potential to be an effective treatment for late‐stage BC, demonstrating antitumour activity, whereas, for early‐stage BC, most of the evidence suggests a pro‐tumour effect. In late‐stage BC, 2‐ME inhibited tumour growth, increased tumour necrosis and slowed pulmonary metastasis. In early‐stage BC, pulmonary metastasis was associated with increased tumour volume and a higher number of CD163+ macrophages. As the effect of 2‐ME on the two BC stages differed, future research should focus on the mechanism and influence of 2‐ME in the TME of the various BC stages for the treatment to be an effective cancer therapy.

## CONFLICT OF INTEREST STATEMENT

The authors declare no conflict of interest.

## Supporting information

Supplementary Fig 1: PCR amplicons. The ruler ladder (L) indicates the sizes of the amplicons. The transgene bands were 556 bp (blue arrow) and the internal positive control bands were 200 bp (**orange** arrow). The numbers 500‐537 were utilized to identify the mice.

## Data Availability

The data supporting the results cited in the text can be found in the relevant articles cited in the references.
