# Supplementary material for: Effect of 2‐methoxyestradiol treatment on early‐ and late‐stage breast cancer progression in a mouse model
Source: Cell Biochem Funct. 2023 Aug 30;41(7):898–911. doi: 10.1002/cbf.3842 (PMC10947225; doi:10.1002/cbf.3842)
Supplement: Supplementary file 1 — Supplementary Fig 1: PCR amplicons. The ruler ladder (L) indicates the sizes of the amplicons. The transgene bands were 556 bp (blue arrow) and the internal positive control bands were 200 bp (orange arrow). The numbers 500‐537 were utilized to identify the mice. [file CBF-41-898-s001.docx]

**Supplementary Fig 1**


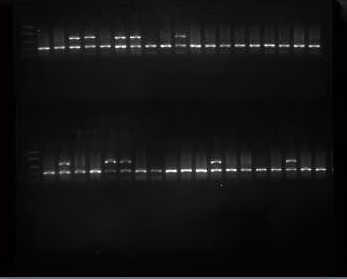


Original Image


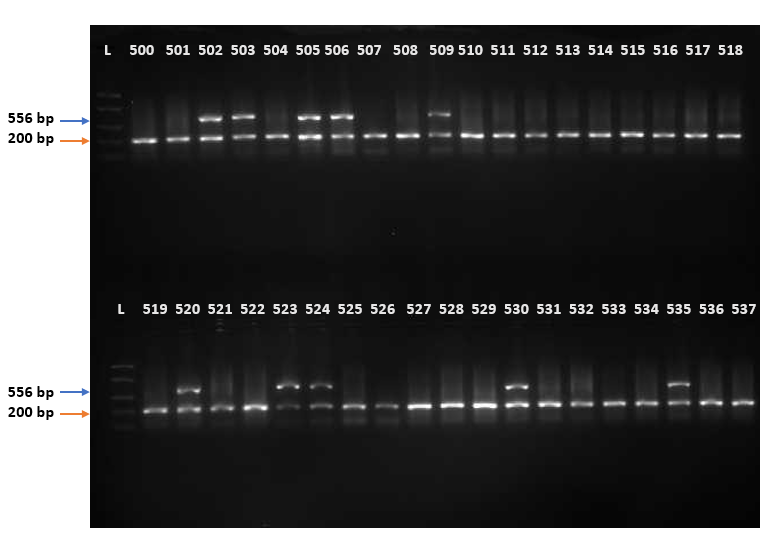


Supplementary Fig 1: PCR amplicons. The ruler ladder (L) indicates the sizes of the amplicons. The transgene bands were 556 bp (blue arrow) and the internal positive control bands were 200 bp (orange arrow). The numbers 500-537 were utilized to identify the mice.
